# Environmental footprint of in-center hemodialysis in Türkiye: A national, scenario-based analysis of water, energy, and waste

**DOI:** 10.1371/journal.pone.0354903

**Published:** 2026-07-30

**Authors:** Berrak Itır Aylı, Mehmet Emin Demir, Arzu Akgül, Hatice Şahin

**Affiliations:** 1 University of Westminster, London, United Kingdom; 2 Department of Nephrology, Medicana International Ankara Hospital, Ankara, Türkiye; 3 Etlik City Hospital Department of Nephrology, Ankara, Türkiye; NED University of Engineering and Technology, PAKISTAN

## Abstract

**Background:**

Hemodialysis (HD) is a life-sustaining but resource-intensive therapy, consuming large volumes of water and electricity and generating substantial waste. This hybrid modeling study quantified the environmental footprint of in-center HD in Türkiye using national registry data, published parameters, and measured facility-level data from two Turkish dialysis centers.

**Methods:**

A hybrid modeling approach combined published per-session resource parameters with measured facility-level data from two Turkish hemodialysis centers (4,000 sessions/month combined). Literature-derived parameters (493 L water/session at 42% RO recovery, 6.2–19.6 kWh electricity/session, 1.5–8.0 kg waste/session) were scaled nationally for 2023 (9.27 million sessions). Measured Turkish data (390 L water, 10.8 kWh electricity, 1.19 kg hazardous waste per session) provided empirical calibration. GHG emissions were calculated using Türkiye-specific (0.494 kg CO_2_e/kWh for electricity) and international conversion factors.

**Results:**

Using literature parameters, annual national estimates include 4.57 million m³ water, 57.5–181.8 GWh electricity, and 13.9–74.2 kt waste, with total GHG emissions ranging from 45.3 to 171.1 kt CO_2_e/year. Measured Turkish facility data yielded lower per-session water consumption (~390 L vs. 493 L, a 21% reduction) and hazardous waste generation (1.19 vs. 1.5 kg/session under good segregation), while electricity consumption (mean 10.8 kWh/session) aligned with the moderate literature scenario. Electricity was the dominant emission source (63–72%), followed by waste incineration (up to 47% with poor segregation).

**Conclusion:**

In-center HD in Türkiye imposes a substantial yet modifiable environmental burden. Measured Turkish facility data demonstrated that actual water consumption is meaningfully lower than international estimates, highlighting the importance of locally validated parameters. Waste segregation and electricity decarbonization represent the highest-impact intervention points for reducing the sector’s carbon footprint.

## Introduction

Hemodialysis (HD) is the predominant modality of kidney replacement therapy (KRT) worldwide. According to the Global Burden of Disease Study 2023, an estimated 4.59 million individuals were receiving KRT globally by 2023, of whom 3.57 million (78%) were on dialysis [1]. The distribution of KRT modalities varies markedly by income level: high-income countries have substantially higher transplantation rates and correspondingly lower proportions of patients on dialysis, whereas in middle- and low-income settings, dialysis — and in-center hemodialysis in particular — remains the dominant therapy. In Türkiye, 71% of all KRT patients receive in-center HD, with peritoneal dialysis accounting for only 5.4% and transplantation for 23.5% of the KRT population [2]. National demand for renal replacement therapy in Türkiye is projected to continue increasing through 2035, driven by rising prevalence of diabetes, hypertension, and population aging [3]. Although life-sustaining for patients with kidney failure, HD is also one of the most resource-intensive treatments delivered routinely within health systems. Each in-center HD session requires substantial volumes of treated water, continuous electricity supply, and generates significant quantities of single-use medical waste. As the global dialysis population continues to grow, these cumulative resource demands have become increasingly relevant to nephrology practice, not only from an environmental perspective but also in terms of health-system sustainability and stewardship.

A standard 4-hour in-center HD session with a dialysate flow of 500 mL/min typically requires 500 L of water. Notably, the majority of input water (approximately 58%) never reaches the patient and is discarded by the reverse osmosis (RO) system as reject despite being near-drinking quality [4,5]. In parallel, each treatment generates considerable plastic waste. A study by Piccoli et al., reported between 1.5 kg and 8 kg of waste per session, depending on the equipment used and on whether waste is properly segregated into non-hazardous vs. biohazard streams [6].

Electricity use in HD is another major concern. Reported energy consumption ranges widely: efficient setups with one RO per machine may use 6.2 kWh per session, whereas units with centralized water systems can consume 12–19.6 kWh per session [7].

Despite these large footprints, accumulating evidence demonstrates that HD’s environmental impact is reducible. Previous studies have shown 52% reduction in water usage per session, 30% reduction in energy use, and 39% reduction in waste generation through monthly resource monitoring and targeted technical upgrades even as dialysis treatment volume doubled [8,9]. Other international centers have pioneered “green dialysis” measures such as reusing RO reject water for hospital cleaning, sanitation, or landscaping; installing solar panels to supply dialysis power; and incentivizing waste minimization and recycling [10]. Furthermore, United Kingdom’s Green Nephrology program, estimated that widespread adoption of sustainability measures in renal units could save the National Health Service up to £1 billion annually [11]. These examples underscore that the environmental footprint of dialysis is actionable: with concerted effort, dialysis services can significantly cut water, energy, and plastic use without compromising patient care [12].

Against this global backdrop, we present a detailed assessment of in-center HD’s environmental footprint in Türkiye, using published resource consumption parameters from conservatively selected peer-reviewed literature, scaled to the national level. We focus on “direct” resource inputs (water, electricity, consumables at point of care) and their associated emissions, recognizing that these are under the immediate control of dialysis providers. We also discuss key intervention points, from high-efficiency RO systems and water reuse strategies to waste segregation and policy changes, that could align Turkish HD practices with international sustainability guidelines [13,14].

To complement the literature-based modeling approach, we obtained measured resource consumption data from two outpatient hemodialysis centers in Ankara, Türkiye, using utility billing records. This hybrid approach, combining published parameters with locally measured foreground data, allows us to assess whether international benchmarks apply to Turkish settings and provides the first empirically calibrated environmental footprint estimate for HD in a middle-income, water-stressed country. Importantly, this analysis is not intended as a full life-cycle assessment of dialysis but as a nephrology-focused evaluation of direct, controllable processes within dialysis units. By concentrating on water treatment, electricity consumption, and waste handling at the point of care, we aim to identify practical intervention points that are immediately actionable for clinicians, unit managers, and policymakers.

## Materials and methods

### Study design and scope

This modeling study estimated point-of-care resource consumption (water, electricity, waste) for maintenance HD in Türkiye. The 2023 Turkish Nephrology Registry reported 9,273,992 outpatient HD sessions [2]; per-session values were scaled linearly to obtain national estimates. Upstream processes (equipment manufacturing, transportation) were excluded. As this study used publicly available aggregate data without individual patient information, ethical approval was not required.

### Measured facility-level data collection

To empirically calibrate the literature-based model, we collected resource consumption data from two private outpatient hemodialysis centers in Ankara, Türkiye. Center A operates approximately 1,500 sessions per month using dedicated on-demand reverse osmosis units. Center B performs approximately 2,500 sessions per month using a centralized water treatment system. For each center, electricity consumption was obtained from commercial electricity invoices, water consumption from utility bills, and hazardous (clinical) waste quantities from licensed medical waste disposal invoice. Data were collected for one billing cycle per center (February–March 2026). These measured values were compared with the literature-derived parameters and used to generate an empirically calibrated national estimate alongside the literature-based model.

### Water consumption per session

Per-session usage estimated at 0.493 m³ (493 L) for 4-hour treatment at 500 mL/min dialysate flow, including 120 L product water, 34 L priming, 51 L rinsing, and ~288 L RO reject. This implies ~42% RO recovery, however, modern high-efficiency RO systems can achieve 70–75% recovery (reducing use to 260–285 L/session), with specialized configurations approaching 90% (228 L/session) [4,5,15]. We retained 493 L as baseline but discuss improvement potential.

### Electricity consumption

Three scenarios captured facility configurations:

- Low-demand: 6.2 kWh/session (dedicated on-demand RO units)
- Moderate: 12.0 kWh/session (central RO, intermittent operation)
- High-demand: 19.6 kWh/session (continuously-running older systems)

These bounds reflect literature-reported ranges for HD energy consumption [7,8]

### Waste generation

Solid waste was estimated at 8.0 kg/session based on Piccoli et al. [6]. Two scenarios modeled handling practices:

- Good segregation: 1.5 kg hazardous (dialyzer/bloodlines incinerated), 6.5 kg municipal waste
- Poor segregation: All 8.0 kg treated as hazardous waste

### Sensitivity analysis

To assess the relative influence of each input parameter on total per-session greenhouse gas emissions, we performed one-at-a-time (OAT) sensitivity analysis. Each parameter, electricity consumption (6.2–19.6 kWh/session), water volume (390–493 L/session, incorporating the measured Turkish lower bound), and waste segregation practice (1.5 vs. 8.0 kg hazardous/session), was varied individually across its full scenario range while holding the remaining inputs at baseline values (moderate electricity at 12.0 kWh, literature water at 493 L, good segregation at 1.5 kg hazardous). The percentage change in total per-session CO_2_e relative to the baseline was calculated to identify dominant emission drivers.

### Greenhouse gas (GHG) emission calculation and example scenarios

To translate resource use into greenhouse gas (GHG) emissions, we applied standard emission factors to the above data. For electricity, Türkiye’s national grid emission factor for 2023 was used as 0.494 kilograms of CO_2_-equivalent per kilowatt-hour (kWh) [16]. This value reflects the average carbon intensity of electricity generation in Türkiye and was obtained from international energy datasets. For water, we applied emission factors for both clean-water supply (treatment and pumping) and wastewater treatment, which together total 0.362 kilograms of CO_2_-equivalent per cubic meter [17]. No Türkiye-specific lifecycle emission factors for water supply or wastewater treatment were available at the time of analysis; accordingly, UK Department for Energy Security and Net Zero (DESNZ) conversion factors were used as the internationally accepted standard for healthcare environmental accounting.

For waste, incineration of clinical waste is highly energy-intensive and produces substantial GHGs, while municipal waste disposal (landfill or recycling) has a much lower direct carbon footprint. Based on a hospital waste lifecycle study, we used 1.074 kilograms of CO_2_-equivalent per kilogram for hazardous (incinerated) waste and 0.004685 kilograms of CO_2_-equivalent per kilogram for municipal waste [18].

The carbon footprint per session was calculated using the following equation:


$$\begin{array}{c} {\text{CO}}_{\text{2}}{\text{e}}_{\text{session}}=(\text{E}\times \text{0}.\text{494})+(\text{W}\times \text{0}.\text{362})+({\text{M}}_{\text{s}}\times \text{0}.\text{004685})+({\text{H}}_{\text{s}}\times \text{1}.\text{074}) \end{array}$$

where E = electricity (kWh), W = water (m³), Ms = municipal waste (kg), and Hs = hazardous waste (kg).

## Results

*All resource consumption values are reported per 4-hour HD session unless otherwise stated; annual national totals (based on 9.27 million sessions in 2023) are provided in parentheses.*

### Water use and wastewater

**Fig 1:**
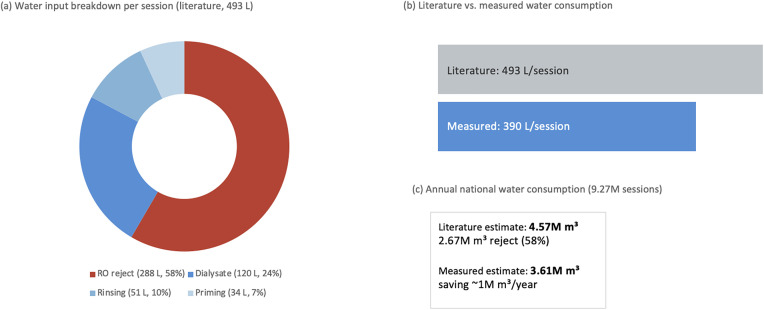
Per-session water consumption in hemodialysis.

Water Consumption Each HD session consumed 493 L of water: 120 L (24%) as dialysate, 85 L (18%) for priming and rinsing, and 288 L (58%) discarded as RO reject (Fig 1). Nationally, total water consumption reached 4.57 million m³/year, of which 2.67 million m³ was reject water. With high-efficiency RO systems achieving 85% recovery, annual water consumption could be reduced by approximately 2 million m³ [19].

### Electricity use and energy footprint

**Fig 2:**
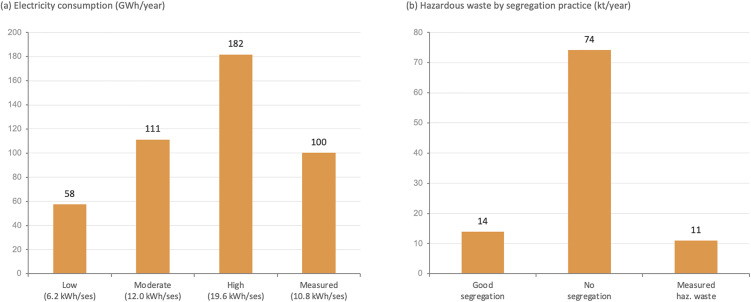
Annual electricity consumption and waste generation by scenario.

Annual electricity consumption ranged from 57.5 GWh (low-demand scenario) to 181.8 GWh (high-demand scenario), with the moderate scenario at 111.3 GWh (Fig 2). Modern energy-efficient systems with adaptive controls could reduce per-session consumption by 15–20%, representing potential national savings of approximately 20 GWh annually [20].

### Waste generation and classification

Using 8.0 kg of total waste per session, Türkiye’s in-center HD treatments would produce roughly 74.192 kilotonnes of solid waste per year if no segregation is practiced (i.e., all waste treated as hazardous). This represents the upper-bound, worst-case scenario. Conversely, under good segregation (only 1.5 kg per session hazardous, the rest general), hazardous clinical waste would total about 13.911 kilotonnes/year (Fig 2). Effective segregation could thus prevent approximately 60.281 kt of waste from unnecessary incineration annually.

### Measured resource consumption from Turkish hemodialysis centers

**Fig 3:**
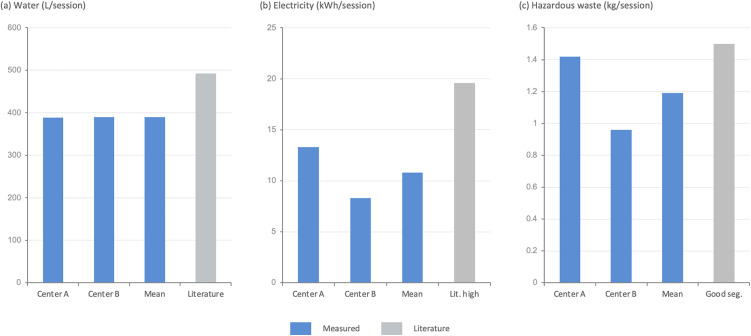
Comparison of measured Turkish facility data with literature-derived parameters.

Table 1 presents the measured per-session resource consumption from two outpatient hemodialysis centers in Ankara, derived from utility billing records. Both centers utilised centralised RO systems and demonstrated remarkably consistent water consumption (~390 L/session), approximately 21% lower than the literature-derived estimate of 493 L/session. This was assumed to be the case because these facilities operate reverse osmosis systems with recovery rates of approximately 50%, higher than the ~ 42% recovery assumed in the literature-based model. Electricity consumption averaged 10.8 kWh/session (range 8.3–13.3), falling within the moderate portion of the published range (6.2–19.6 kWh/session). Centre A, which had machines ranging from 7 to 19 years old from three different providers, consumed 13.3 kWh/session, while Centre B, with all machines less than 10 years old, consumed 8.3 kWh/session; both centres operated the same RO system. Measured hazardous waste generation averaged 1.19 kg/session (range 0.96–1.42), close to the 1.5 kg/session assumed under good segregation in the literature model; total waste per session (including municipal waste) was not captured by records. Comparisons with the literatüre have been illustrated in Fig 3.

**Table 1:** Measured per-session resource consumption from two Turkish hemodialysis centers (Ankara, 2026) compared with literature parameters.

Parameter	Center A	Center B	Mean	Literature
Electricity (kWh/session)	13.3	8.3	10.8	6.2–19.6
Water (L/session)	389	390	390	493
Hazardous waste (kg/session)	1.42	0.96	1.19	1.5–8.0¹
GHG per session (kg CO_2_e)	8.24	5.27	6.76	4.88–18.45²

^1^Total waste range; hazardous component is 1.5 kg under good segregation. ^2^Full range across all electricity and waste scenarios. GHG for measured centers calculated using measured hazardous waste only; municipal waste not captured by billing records.

When the measured Turkish values were applied to the national model, estimated annual water consumption was 3.61 million m³ (compared with 4.57 million m³ using literature parameters), electricity consumption was 100.3 GWh/year, and hazardous waste was 11.0 kt/year. The corresponding national GHG estimate using measured values was 62.7 kt CO_2_e/year, which falls within the lower-to-middle portion of the literature-based range (45.3–171.1 kt CO_2_e/year) and is closest to the moderate-electricity, good-segregation scenario (71.9 kt CO_2_e/year), suggesting that well-operated Turkish centers are achieving environmental performance consistent with moderate-efficiency international benchmarks.

### Carbon emissions and hotspots

**Fig 4:**
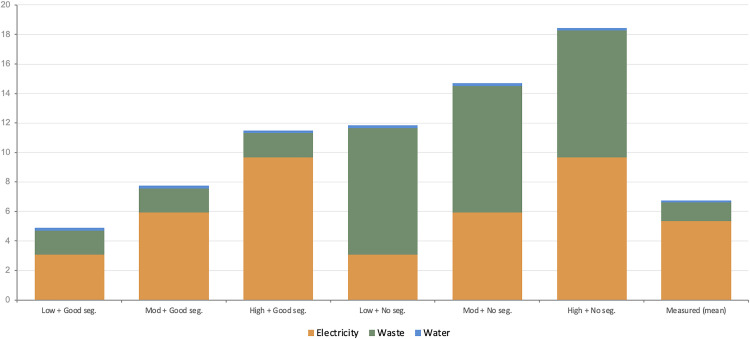
Greenhouse gas emissions per session by source and scenario.

Per-session emissions ranged from 4.9 kg CO_2_e (best case: efficient systems, good segregation) to 18.5 kg CO_2_e (worst case: inefficient systems, poor segregation) (Table 2). Annual national emissions ranged from 45.3 to 171.1 kt CO_2_e. Electricity was the dominant contributor (63–72% of total emissions), followed by waste handling (up to 47% with poor segregation). Water-related emissions contributed less than 4% across all scenarios. Proper waste segregation alone could avoid approximately 64 kt CO_2_e/year nationally. Fig 4 presents a summary overview comparing per-session resource consumption and GHG emissions across the literature-based and measured Turkish scenarios.

Sensitivity analyses indicated that per-session emissions were most sensitive to electricity consumption assumptions (±30% impact) and waste segregation practices (±47% impact), while water-related parameters had minimal influence (±3%).

**Table 2:** Greenhouse gas emissions per session and annual national totals by scenario.

Electricity Scenario	Waste Handling	Elec. CO_2_e	Water CO_2_e	Waste CO_2_e	Total (kg CO_2_e/session)	Annual (kt CO_2_e/yr)
Low (6.2 kWh)	Good segregation	3.06	0.18	1.64	4.88	45.3
Moderate (12.0 kWh)		5.93	0.18	1.64	7.75	71.9
High (19.6 kWh)		9.68	0.18	1.64	11.50	106.7
Low (6.2 kWh)	No segregation	3.06	0.18	8.59	11.83	109.7
Moderate (12.0 kWh)		5.93	0.18	8.59	14.70	136.3
High (19.6 kWh)		9.68	0.18	8.59	18.45	171.1

Values in kg CO_2_e/session except Annual column (kt CO_2_e/year). Electricity emission factor: 0.494 kg CO_2_e/kWh (Türkiye 2023 grid). Water: 0.362 kg CO_2_e/m³. Good segregation: 1.5 kg hazardous + 6.5 kg municipal. No segregation: all 8.0 kg as hazardous.

## Discussion

This hybrid modeling study provides the first national-scale estimate of in-center hemodialysis’s environmental footprint in Türkiye, combining published resource parameters with measured facility-level data from two Turkish dialysis centers. The analysis identified electricity consumption and waste handling as the primary determinants of greenhouse gas emissions, while water consumption, though volumetrically substantial, contributed minimally to the carbon footprint. Notably, measured water consumption in Turkish facilities (~390 L/session) was approximately 21% lower than the literature estimate (493 L/session), suggesting that modern RO systems in Turkish centers achieve moderately higher recovery rates (~50%) than assumed in published models derived from older international data (~42%). To contextualize the overall impact, the estimated 45–171 kt CO_2_e/year from in-center HD in Türkiye is equivalent to the annual emissions of approximately 10,000–37,000 passenger cars, or the annual electricity consumption of 26,000–99,000 Turkish households. The 4.57 million m³ of water consumed annually under literature parameters would fill approximately 1,800 Olympic-sized swimming pools, of which over 1,000 pools’ worth is discarded as RO reject.

### Water management

Our results highlight the outsized water requirements of HD and reinforce that improving water efficiency is critical for long-term sustainability. The fact that ~58% of input water is routinely wasted calls for a systematic rethink of dialysis water systems. High-efficiency RO technology is one immediate solution. As noted, leading manufacturers have developed dialysis water systems capable of 75–85% recovery, with some models advertising up to 90% permeate yield via advanced recirculation and volume-management features [19–22]. Widespread adoption of such systems could drastically cut water consumption by HD units. However, it is essential to validate performance in real-world conditions: recovery rates may be lower in practice and pushing RO to extreme efficiency can risk membrane fouling [23]. Studies have confirmed that aiming for 100% recovery is impractical, it significantly increases energy use and shortens membrane life, ultimately negating the benefits. Therefore, moderate recovery optimization (e.g., achieving 70–80% reliably) combined with reuse of the remaining reject water is the recommended strategy [9,24].

Our findings indicate that approximately 2.6 million cubic meters of reject water in Türkiye are potentially reusable each year. In Australia and Morocco, dialysis reject water from satellite units has been used for irrigiation of public areas [25,26]. Turkish health authorities could issue protocols or incentives for dialysis providers to implement reuse projects, especially in water-stressed regions.

### Measured vs. Modeled resource consumption

The incorporation of directly measured Turkish facility data revealed important divergences from literature-based estimates. The most notable finding was the consistency of water consumption across both surveyed centers (~390 L/session), approximately 21% below the literature value of 493 L/session. This suggests that modern private dialysis facilities in Türkiye operate RO systems with recovery rates exceeding the 42% assumed in published international models. While the measured reduction is more moderate than might be achieved with the latest high-efficiency equipment (which can reach 75–90% recovery), it demonstrates that even standard modern systems outperform the literature baseline. If this finding is generalizable, literature-based national water consumption estimates would overstate actual use by approximately one-fifth.

Electricity consumption in the measured centers (mean 10.8 kWh/session) aligned closely with the moderate literature scenario (12.0 kWh/session). Centre A, with older machines from 3 different providers, consumed 13.3 kWh/session, while Centre B from a, with newer ones from a single provider, consumed 8.3 kWh/session. This within-sample variation illustrates how individual differences between centres influence energy demand and underscores the importance of system-level choices in determining environmental performance. Hazardous waste generation (mean 1.19 kg/session) was close to the 1.5 kg/session “good segregation” benchmark, suggesting that the surveyed centers practice effective waste segregation. Differences between Turkish and European consumable sizes and packaging practices may also contribute to the slightly lower measured values.

These findings underscore the value of locally measured data for environmental assessments in healthcare. National estimates based solely on international parameters risk overestimation (as with water) or may not capture the heterogeneity of real-world facilities. We recommend that future studies incorporate multicenter measurement campaigns across diverse facility types, including public hospitals and university centers with potentially older equipment.

### Energy efficiency and renewable power

The carbon footprint analysis identified electricity as the largest contributor to HD emissions, accounting for 63–72% of total GHG across scenarios. Thus, decarbonizing dialysis will require reducing electricity use and/or greening its source. On the demand side, dialysis machines often run at fixed dialysate flow (e.g., 500 mL/min) even when patient clearance needs would be met with less. Studies suggest that reducing dialysate flow in appropriate scenarios (or using intelligent flow scaling) can lower water and energy use without impacting treatment efficacy [27]. Likewise, ensuring RO units are not running or recirculating water when the dialysis ward is closed (nights, off-days) can save significant energy. Automated controls to shut off or idle systems when not needed are increasingly available and should be utilized [28].

Replacing ageing RO systems and dialysis machines with newer models can yield large efficiency gains through features like variable-frequency drives, heat disinfection, and smart feedback to minimize wasteful operation [19,23]. While capital-intensive, such upgrades often pay for themselves through water and electricity savings over the equipment lifespan. Government or green healthcare grants could help centers invest in these technologies. Some HD centers have implemented regenerative systems, such as recovering heat from warm dialysate effluent to pre-warm incoming water, yielding 10–20% energy savings, and on-site battery storage [7,28,29].

Ultimately, powering dialysis with renewables can drastically cut its carbon emissions. On-site solar-photovoltaic systems have been successfully deployed in international examples, offsetting up to 70–90% of grid electricity use per HD unit [4,7]. In Türkiye, many dialysis centers are physically well-suited for rooftop solar arrays, and purchasing certified green electricity from the grid could further decarbonize operations. Our findings imply that even a 15–20% reduction in per-session energy use (attainable via modern RO pumps and practices) would cut the national HD carbon footprint by on the order of 10 kt CO_2_e/year, and moving to 100% renewable electricity for dialysis could eliminate 50–100 kt CO_2_e/year.

### Waste reduction and circular economy

The other major lever for sustainability in HD is waste management. Our analysis demonstrated that how dialysis waste is handled can have as significant an impact on GHG emissions as electricity consumption, since incinerating plastic is highly carbon-intensive and HD generates substantial quantities of single-use materials [6,8]. Therefore, strict waste segregation remains one of the simplest and most cost-effective interventions to improve environmental performance. The measured hazardous waste data from the two Turkish centers (mean 1.19 kg/session) suggest that effective segregation is already being practiced, with values close to the 1.5 kg/session international benchmark. Dialysis unit staff should be trained and periodically retrained on what constitutes infectious waste and what does not, as staff education alone has been shown to reduce hazardous waste significantly, and further recycling initiatives for non-contaminated plastics offer additional areas for mitigation [6,29,30].

### Policy and system-level recommendations

To catalyze the above changes, top-down support from healthcare authorities and professional bodies is essential. At present, no Turkish regulation explicitly mandates environmental performance criteria for dialysis centers. Some providers may act voluntarily, but widespread implementation will likely require external drivers and standardized expectations.

- Incorporate sustainability into standards and accreditation: Dialysis unit licensing or accreditation criteria (by the Ministry of Health or independent agencies) should include indicators of resource efficiency. For example, units could be required or incentivized to report water consumption (L/session) or percentage of hazardous waste as part of quality assurance frameworks. National standards could encourage high water recovery rates and low specific energy consumption. Professional bodies such as the Turkish Society of Nephrology could further reinforce standards by issuing green dialysis certification criterias.
- Promote data transparency and eco-reporting: As demonstrated by the 13-year French “eco-dialysis” initiative, simply measuring and disclosing energy, water, and waste metrics drives continuous improvement [29]. Implementing a national eco-reporting program that tracks water, electricity, and hazardous-waste proportion could enable benchmarking across centers. Facilities with high footprints could then receive targeted technical support. This system could be integrated into the Turkish Nephrology Registry; and global efforts could perhaps be reported in the European Renal Associations registries.
- Economic incentives and financial support: Policymakers and payers can accelerate adoption through fiscal mechanisms. An example would be capital grants, tax breaks or low-interest loans for centers upgrading to high-recovery RO units or installing rooftop solar systems. Since clinical-waste incineration costs are several times higher than those of municipal disposal [18], dialysis units already have an economic rationale for improving segregation; policy alignment and country wide education in centers for better practice can amplify this incentive.
- Adopt WHO and international recommendations: The World Health Organization’s Operational Framework for Building Climate-Resilient and Low-Carbon Health Systems (2021) highlights water efficiency, waste minimization, and energy transition as core actions for all health facilities [31]. Türkiye’s health authorities could formally include dialysis within these commitments. Clinicians should also be familiarised with the European Renal Association (ERA) Green Nephrology Good Practice Guide (2023) and other global resources that synthesise practical sustainability interventions [32]. To close knowledge gaps, sustainability content should be integrated into nephrology curricula, national conference workshops, and ongoing professional development programs.

### Limitations

Several limitations warrant consideration. First, per-session resource estimates from the literature were derived from published international studies rather than direct measurement in Turkish facilities; actual consumption may vary across centers. Second, UK-derived emission factors were used for water and waste processes due to the lack of Türkiye-specific lifecycle data. If Turkish waste incineration is more carbon-intensive than UK processes, our estimates may underestimate actual emissions; however, the relative contribution rankings (electricity > waste > water) are likely robust. A recent lifecycle assessment of medical waste management in Istanbul confirmed that incineration-dominant scenarios carry the highest environmental burden [33], supporting the directionality of our findings. Third, our boundary excluded manufacturing, supply chain, and patient transportation impacts. Finally, as a cross-sectional estimate, this analysis does not account for projected growth in dialysis demand. Türkiye, like most countries, is seeing rising dialysis patient numbers annually; without interventions, the resource use and emissions will only increase.

The measured facility data are drawn from only two private outpatient centres in a single city (Ankara) and may not represent the full diversity of Turkish HD facilities, which include university hospitals, public hospitals, and centres with varying equipment ages. Additionally, the centre records provide whole-facility resource use rather than dialysis-specific metering, meaning that some non-dialysis loads (e.g., administrative areas, staff facilities) may be included in the electricity and water figures, potentially overestimating per-session consumption. Municipal (non-hazardous) waste was not recorded in billing data and therefore remains estimated from the literature. Despite these limitations, the convergent water consumption values across both centers (~390 L/session) provide reasonable confidence that these measurements reflect a genuine pattern in modern Turkish dialysis facilities.

## Conclusion

This study provides the first national-scale environmental footprint assessment for in-center hemodialysis incorporating measured Turkish facility data. Three findings with immediate practical implications emerged. First, measured water consumption in Turkish centers (~390 L/session) was approximately 21% lower than the internationally published estimate (493 L/session), indicating that modern facilities achieve moderately higher RO recovery rates than commonly assumed; literature-based models should be validated against local measurements before informing policy. Second, waste segregation represents the single highest-impact, lowest-cost intervention: enforcing proper segregation so that only contaminated materials are incinerated could avoid an estimated 60–65 kt CO_2_e annually at the national level. Third, electricity is the dominant emission source, contributing 63–72% of total GHG emissions per session; decarbonization through equipment efficiency upgrades and renewable energy integration offers the largest long-term emission reduction potential.

These findings support the integration of environmental performance metrics, water consumption per session, electricity intensity, and hazardous waste proportion, into routine quality assurance frameworks for dialysis centers. Türkiye’s renal services can align with international Green Nephrology strategies while generating cost savings through reduced water, energy, and waste disposal expenses. Future multicenter studies with direct resource metering across diverse facility types will be essential to refine these estimates and monitor progress.

## Supporting information

S1 File Graphical Abstract. (PPTX)
