# Supplementary material for: Environmental footprint of in-center hemodialysis in Türkiye: A national, scenario-based analysis of water, energy, and waste
Source: PLoS One. 2026 Jul 30;21(7):e0354903. doi: 10.1371/journal.pone.0354903 (PMC13422879; doi:10.1371/journal.pone.0354903)
Supplement: S1 File — (PPTX) [file pone.0354903.s001.pptx]

## Slide 1
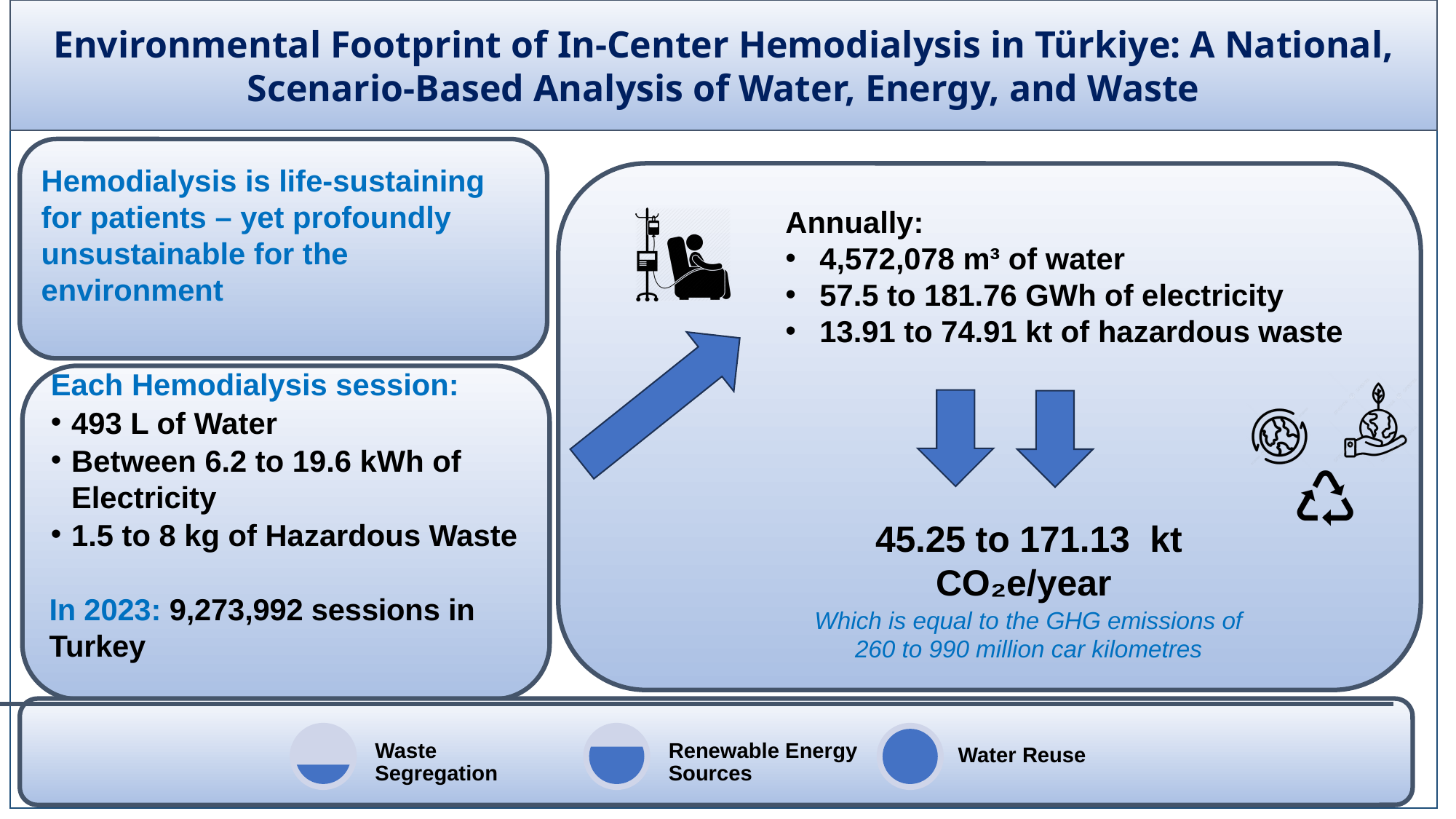

Environmental Footprint of In-Center Hemodialysis in Türkiye: A National, Scenario-Based Analysis of Water, Energy, and Waste
Hemodialysis is life-sustaining for patients – yet profoundly unsustainable for the environment
Annually:
4,572,078 m³ of water
57.5 to 181.76 GWh of electricity
13.91 to 74.91 kt of hazardous waste
Each Hemodialysis session:
493 L of Water
Between 6.2 to 19.6 kWh of Electricity
1.5 to 8 kg of Hazardous Waste
In 2023: 9,273,992 sessions in Turkey
45.25 to 171.13 kt CO₂e/year
Which is equal to the GHG emissions of 260 to 990 million car kilometres
